# Doctors Rule: An Analysis of Health Ministers’ Diaries in Australia

**DOI:** 10.3390/ijerph16132440

**Published:** 2019-07-09

**Authors:** Katherine Cullerton, Tom White, Amanda Lee

**Affiliations:** 1 School of Public Health, Faculty of Medicine, University of Queensland, 288 Herston Road, Herston, QLD 4006, Australia; 2 MRC Epidemiology Unit, University of Cambridge School of Clinical Medicine, Box 285, Institute of Metabolic Science, Cambridge Biomedicine Campus, Cambridge CB2 0QQ, UK

**Keywords:** nutrition policy, advocacy, food industry, public health, health policy, lobbying, policy making

## Abstract

Limited progress in nutrition policy action is often blamed on the close relationships the food industry has with health policy decision-makers. This analysis sought to examine this belief through the analysis of health ministers’ diaries. Entries were downloaded from health ministers’ diaries from two states in Australia from January 2013 to June 2018. Entries were coded according to which interest group met with the minister or whether general parliamentary business was undertaken. Coding was also undertaken for any meeting topics related to nutrition policy. Analysis of health ministers’ diaries found that the food industry has limited documented interaction with the two state health ministers in Australia. Instead, medical associations, private hospitals and health services, and sporting associations (rugby league associations) had the most interactions with health ministers. Poor representation was seen on nutrition issues, and there was an apparent lack of nutrition advocates interacting with the health ministers. There are opportunities for nutrition advocates to increase their level of interaction with state health ministers. This could include building alliances with medical associations, as they are in a powerful position, to advocate directly to health ministers. Health ministers’ diaries can provide valuable insights into who is meeting officially with ministers. However, there are also limitations with the dataset.

## 1. Introduction

There are many ways interest groups can attempt to influence public policy. Strategies can include engaging with the media, shaping the evidence base, making donations to political parties and grassroots campaigns [1,2]. While these strategies are important, direct access to policymakers seems to play a significant role in influencing public policy, particularly for policy that is contested [3,4]. Gaining direct access to policymakers allows interest groups to develop relationships with them, deliver their arguments more effectively and identify potential policy leverage points [2].

A particularly contested health policy area in many countries is public health nutrition policy [5,6]. This has certainly been the case in Australia, where, over the past decade, there has been a distinct lack of political support for evidence-based nutrition policy actions, such as fiscal and regulatory interventions [4,7]. Instead, the government has supported education-based campaigns and voluntary initiatives involving the food industry [8]. It has been proposed that this inaction in nutrition policy is due to the opposing power of food industry interest groups [1,7,9]. The power and influence of the food industry was recently demonstrated in a network analysis of national nutrition policy stakeholders in Australia [4]. This analysis highlighted that the food industry had the greatest number of direct access points to nutrition policymakers than any other interest group. However, limited investigation of interest groups and their interactions with policymakers has occurred at the state jurisdictional level in Australia.

Examining the interactions of interest groups at a state level is important, as Australia is a federation of six states and two self-governing territories, each of which have their own constitutions and laws. However, certain areas of law-making require both the state and national levels of government to work together to achieve policy outcomes [7]. This can give states a high degree of power when considering nutrition policy.

In January 2013, the state of Queensland (QLD) was the first jurisdiction in Australia to require government ministers to release their diaries on a monthly basis [10] and the state of New South Wales (NSW) followed in July 2014, although with quarterly releases [11]. While this was an important step toward transparency, in both jurisdictions, there is no requirement to disclose information relating to personal, electorate or party-political matters, social or public functions or events, or matters for which there is an over-riding public interest against disclosure [10,11].

The diaries are available for two jurisdictions which represent just over half of the Australian population (NSW: 7,955,900; QLD: 4,999,700) [12]. Further information on these states and the political parties in Australia is included in Box 1. Analysing the health ministers’ diaries from these two states provided unique insight into which interest groups were interacting with health ministers generally, and in particular, with respect to nutrition policy.

Box 1 **The jurisdictions of New South Wales and Queensland.**

**The jurisdictions of New South Wales and Queensland.**

New South Wales is considered the most powerful state in Australia, as it has the largest population and economy [13]. Furthermore, it is a manufacturing state and many national and international companies have their headquarters located there. QLD is known for agriculture, mining and tourism and is the third wealthiest state in Australia [13]. Since ministerial diaries have been recorded, QLD has had a change of governing political party, going from the Liberal-National Party (centre-right liberal conservative) to the Australian Labour Party (centre-left), whereas in NSW the Liberal Party (centre-right liberal conservative) has been the governing party throughout. The changes in political party and ministers are included in the table below (Table 1). During this period, NSW appointed an Assistant Health Minister to support the Health Minister. Assistant Ministers are appointed to support Ministers in prioritising work to facilitate public access to the executive and to enable the bureaucracy to have an ongoing point of contact [14]. They do not attend executive council or cabinet meetings. This position was abolished in NSW in January 2017.

**Table 1:** Changes in health ministers in New South Wales and Queensland (2013–2018).

	2013	2014	2015	2016	2017	2018
QLD Health Min	L. Springborg ^a^	L. Springborg ^a^	C. Dick ^b^	C. Dick ^b^	C. Dick ^b^	S. Miles ^b^
NSW Health Min		J. Skinner ^a^	J. Skinner ^a^	J. Skinner ^a^	B. Hazzard ^a^	B. Hazzard ^a^
NSW Assistant Health Min		J. Rowell (also Minister for mental health) ^a^	P. Goward (also Minister for mental health, women & medical research) ^a^	P. Goward (also Minister for mental health, women & medical research) ^a^		

^a^ Centre-right liberal conservative political party (LP or LNP); ^b^ Centre-left political party (ALP).

## 2. Materials and Methods

Ministers’ diaries from January 2013 to June 2018 were downloaded as PDFs from the relevant government websites and data were extracted into an excel spreadsheet. Diary entries were coded according to which interest group was meeting with the minister or if general parliamentary/portfolio business was undertaken. These were broadly classified into four categories:

- *advocacy*: including not-for-profit groups, charities, citizen groups and professional associations,
- *business*: including for-profit organisations, businesses and their peak bodies,
- *university*: public universities or public research institutions, and
- *general business*: any standard parliamentary/portfolio business, community electorate meetings, etc.

Several entries were documented as ‘business receptions’; for the QLD ministers, the diaries generally detailed each company who came to these receptions. When 25 companies or fewer were present, each company was coded individually. When more than 25 companies attended these events, they were coded as ‘general business receptions’, and companies were not coded individually, as potential one-on-one time with the minister would be decreased.

Once each diary entry was coded individually and within the four categories above, the individual entries were collated into general codes around their professional focus, for example, bank, food company, medical association, and cancer organisation. This coding framework was applied to all the diary entries. The initial coding and development of the coding framework was conducted by one investigator and then discussed with the co-authors until consensus was reached. The first author then deductively coded all the diary entries using the agreed framework. Coding was also undertaken for meeting topics specifically around nutrition. Ten percent of the diary entries were double-coded by an independent research assistant.

## 3. Results

In total, 5025 diary entries were coded. QLD health ministers’ diaries covered a period of 63 months and included 3926 diary entries. NSW health ministers’ diaries covered 48 months and included 586 diary entries, while the diaries of the Assistant Health Minister for NSW covered 31 months and included 513 diary entries. The two jurisdictions record diary entries differently, with the QLD ministers’ diaries recording all appointments including general business, and the diaries of the NSW Health Minister and Assistant Minister including limited records of these appointments. To ensure comparability, appointments related to general business were removed. However, it is interesting to note that in the QLD ministers’ diaries, the majority of entries related to general portfolio and electoral business, whereas meetings with interests groups made up only 27% of all diary entries.

The average number of appointments ministers spent with the different interest groups per month can be seen in Table 2.

According to the diaries, the QLD health ministers, regardless of political party, spent considerably more time interacting with advocacy and business groups than the NSW Health Minister. This is even more pronounced for the ALP QLD Health Minister who, on average, interacted with advocacy groups and business representatives almost twice as frequently as the NSW Health Minister.

### Doctors Rule

For advocacy groups, it was clear that across political parties and states, medical associations were dominant in interactions with health ministers. This was particularly evident for the ‘doctors’ union’, the Australian Medical Association (AMA) (Table 3). Rates of interactions between medical associations and the ministers were higher than any other group. Other medical associations that were highly represented across all ministers’ diaries were the Rural Doctors Association (*n* = 23), the Royal Australasian College of Surgeons (*n* = 16) and the Royal Australian and New Zealand College of Psychiatrists (*n* = 12).

For business groups, private hospitals topped interactions with the QLD Health Minister when the LNP were in power (Table 4). In NSW, private health care services had the highest number of interactions with the Health Minister, followed by private hospitals. Conversely, state and national associations representing a popular code of football in Australia (Rugby League) had the greatest interaction with the QLD Health Minister when Labour was in power.

Very few interest groups met with the health ministers specifically regarding nutrition policy issues (Table 5). Of the 5,025 interactions documented, only 16 related specifically to nutrition issues and three of those included no interest group representation, instead involving only the Health Minister and departmental staff. Only one interaction from the food industry (Australian Beverages Council) was recorded regarding a specific nutrition policy issue.

## 4. Discussion

The data in this study were obtained from unique datasets that had not been previously systematically analysed. It is likely, however, that the diaries we have analysed do not capture all the interactions that ministers undertake and we have no way of knowing how closely the diaries represent a minister’s actual day. The level of detail provided in QLD provides a more complete representation than in NSW, where diaries often have days with no entries at all (*n* = 609 days, 64% of NSW health ministers’ days). However, valuable insights are still likely to be gained from the 5025 interactions that were recorded.

### 4.1. The Food Industry Is Poorly Represented

This analysis was originally undertaken to identify whether specific interest groups were engaging regularly with state health ministers, particularly around nutrition policy issues. Despite previous evidence in Australia [4] and internationally [15,16] highlighting direct and frequent engagement of the food industry with health ministers, this was not found to be the case in the two Australian state jurisdictions in this study. There may be several reasons for this, including the limited reporting requirements for ministerial diaries (particularly for NSW), which do not capture after-hours activities, informal meetings on the phone or in person, or who ministerial advisors are meeting with. Furthermore, these groups may be meeting with more senior ministers, for example, the state premier or treasurer, or with other ministries related to food—such as agriculture or trade—or with government bureaucrats. Finally, responsibility for many aspects of nutrition policy sits with the Australian government, so relationship building may be directed there. However, any significant decisions that need to be made around regulation or legislation in Australia require agreement between the Australian government together with all the states and territories, so it is surprising that more interactions were not noted. Alternatively, this lack of representation may also indicate that the ministers’ diaries are not a reliable source for documenting interactions with the food industry.

### 4.2. Advocacy Organisations Are Leading Engagement

The results demonstrate high rates of interaction between ministers and advocacy organisations in comparison to business interests. This differs from the previous research conducted on this issue. Studies from the United States of America demonstrated that the majority of advocacy organisations do not engage in ‘lobbying’ [17]. While this study does not examine the overall proportion of advocacy organisations participating in interactions with Ministers, the higher proportion of meetings by these organisations in comparison to the business sector may signify a change in practice over time. It may also represent a willingness from ministers to engage more widely with advocacy organisations.

Medical associations, particularly the Australian Medical Association, had the greatest number of interactions with ministers. This indicates not only a high level of activism by medical associations, but also a high level of prioritisation of the medical profession by the ministers. This prioritisation corresponds with the traditional view that medicine sits at the top of the occupational hierarchy in health and is considered the cultural authority on health and illness [18]. The observed dominance and influence of the medical profession is not unique to Australia, with several international studies reporting a similar phenomenon [19,20,21,22].

Very few interest groups met with the health ministers specifically regarding nutrition issues. This lack of engagement by nutrition professionals and not-for-profit groups advocating for nutrition issues corresponds with previous research documenting the lack of direct contact with decision-makers in nutrition policy in Australia [4]. This lack of engagement could represent a lack of understanding of the policymaking process and the key role ministers play, and/or a lack of capacity from nutrition advocates in terms of time, or advocates could be prioritising targeting national ministers instead of state-based ministers [23]. However, it is important to note that interest groups may be meeting with the ministerial advisers regarding these issues, and there is no requirement to document such interactions.

### 4.3. Market Solutions to Healthcare?

For business groups, private health care services and private hospitals topped the interactions of the NSW Health Minister and the QLD Health Minister when the LNP were in power. This may signify the growing trend towards finding market solutions to healthcare, a movement that is occurring world-wide [24]. The high level of interactions with private hospital companies coincides with a growth in private hospital beds and, accordingly, government funding for private hospitals in Australia [25]. A very different approach was taken by the QLD ALP health ministers, where rugby league associations had the greatest interaction. These meetings corresponded with additional QLD government funding for the rugby league, including: AUD$1,000,000 to the National Rugby League State of Mind program (designed to reduce stigma around mental illness through rugby league clubs) in 2016, AUD$637,500 over two years for the improvement of rugby league facilities around QLD in 2017, and AUD$165,000 for a children’s rugby league program run by ex-players, also in 2017 [26,27]. Alternatively, these interactions may signify an awareness that the core constituents of the Labour party in QLD are working class and traditionally follow the game of rugby league, so it may be an important political strategy for the Minister to align with this popular code of football.

One final observation relates to the diary entries of the NSW Assistant Health Minister. This position had a very different range of interactions compared to the health ministers in QLD and NSW, most notably high levels of engagement with mental health organisations, homelessness charities and drug and alcohol charities. This increased engagement coincided with the Assistant Minister being named Minister for Mental Health and then also Minister for Women and Medical Research. This suggests that providing specificity in the ministerial title may result in higher levels of engagement with relevant interest groups than if the title is broadly ‘health’.

## 5. Conclusions

We undertook this study to identify which interest groups were engaging with state health ministers in Australia, particularly around nutrition policy. Previous research suggested that the food industry would be a strong presence, but this was not the case in this study. Instead, advocates from the medical profession dominated health ministers’ documented diary time, and from the business side, private hospitals and rugby league associations dominated. Poor representation was seen on nutrition issues, and there was an apparent lack of nutrition advocates interacting with the health ministers. While the lack of documented interactions with the food industry raises questions regarding the completeness of the diaries, the findings do provide valuable insight for a progressing nutrition policy. Opportunities exist for nutrition advocates to increase their level of interaction with state health ministers. This could involve building alliances with medical associations, as they are in a powerful position to advocate directly to health ministers, and there are opportunities to collaborate on mutually shared prevention issues. Ministerial diaries offer a unique dataset which, despite limitations, are important to continue to monitor. To improve insights around lobbying further datasets, political donations, for example, could be combined with this information.

## Figures and Tables

**Table 2:** Average numbers of recorded appointments with interest groups in ministers’ diaries, per month (January 2013–June 2018).

Minister	Time Period	Advocacy (Number of Appointments/Month)	Business (Number of Appointments/Month)	Universities (Number of Appointments/Month)
Health Minister QLD (LNP)	24 months	9.6	5.5	1.3
Health Minister QLD (ALP)	39 months	11.3	5.1	1.3
Health Minister NSW	48 months	6.0	4.1	1.8
Assistant Health Minister NSW	31 months	10.7	2.6	1.7

**Table 3:** Top recorded advocacy interactions in health ministers’ diaries (January 2013–June 2018) *.

Health Minister QLD (LNP) 24 Months	Health Minister QLD (ALP) 39 Months	Health Minister NSW (LP) 48 Months	Assistant Health Minister NSW (LP) 31 Months
Medical association (*n* = 62, AMA = 28)	Medical association (*n* = 56, AMA = 24)	Medical association (*n* = 68, AMA = 21)	Mental health organisations (*n* = 53)
Mental health organisations (*n* = 15)	Workers unions (*n* = 36)	Cancer organisations (*n* = 28)	Homelessness organisations (*n* = 40)
Indigenous organisations (*n* = 13)	Indigenous organisations (*n* = 35)	Citizen groups (*n* = 17)	Drug & alcohol organisations (*n* = 36)
Cancer organisations (*n* = 11)	Nurses’ union (*n* = 28)	Workers unions (*n* = 16)	Domestic violence organisations (*n* = 20)
Nurses’ union (*n* = 11)	Mental health organisations (*n* = 19)	Nurses’ union (*n* = 10)	Community services charities (*n* = 18)
Citizen groups (*n* = 10)	Youth organisations (*n* = 16)	Paramedics (*n* = 10)	Medical (*n* = 15, AMA = 2)
Emergency Medicine Foundation (*n* = 6)	Multicultural organisations (*n* = 14)	Children/youth charities (*n* = 10)	Anti-windfarm groups or individuals (*n* = 14)
Hospital Foundation (*n* = 6)	Citizen groups (*n* = 13)	Heart disease organisations (*n* = 7)	Council of Social Services (*n* = 13)
Royal Flying Doctors Service (*n* = 6)	Hospital Foundations (*n* = 13)	Indigenous organisations (*n* = 6)	Indigenous organisations (*n* = 8)
Workers unions (*n* = 6)		Disability organisations (*n* = 6)	

* *n* = total number of advocacy-related diary entries a Minister had during the period of time they held a health portfolio.

**Table 4:** Top recorded business interactions in health ministers’ diaries (January 2013–June 2018) *.

Health Minister QLD (LNP) 24 Months	Health Minister QLD (ALP) 39 Months	Health Minister NSW (LP) 48 Months	Assis Health Minister NSW (LP) 31 Months
Private Hospitals (*n* = 16)	Rugby League (*n* = 17)	Private health care services (*n* = 28)	Pharmaceutical companies (*n* = 15)
Aged Care (*n* = 9)	Global accounting/consulting firms (*n* = 13)	Private Hospitals (*n* = 22)	Small consultancy/investment firms (*n* = 10)
Infrastructure company (*n* = 9)	Pharmacy guild (*n* = 12)	Medical devices (*n* = 15)	Private disability/mental health companies (*n* = 6)
Biotech company (*n* = 6)	Property company (*n* = 11)	Pharmaceutical companies (*n* = 11)	Construction/building companies (*n* = 5)
Property company (*n* = 6)	General business receptions (*n* = 10)	IT companies (*n* = 8)	Rugby League (*n* = 3)
Private health care services (*n* = 5)	Biotech company (*n* = 8)	Pharmacy guild (*n* = 8)	Clean energy companies (*n* = 3)
Construction company (*n* = 5)	Taiwan business representatives (*n* = 8)	Global accounting/consulting firms (*n* = 8)	Marketing/communication companies (*n* = 3)
Global accounting/consulting firms (*n* = 5)	Aged Care (*n* = 7)	Banks (*n* = 7)	Chamber of Commerce (*n* = 3)
Mining companies (*n* = 4)	Law firms (*n* = 7)	Law firms (*n* = 7)	Small business owner (*n* = 3)
3rd party lobbyists (*n* = 4)	Pharmaceutical companies (*n* = 7)	Finance companies (*n* = 7)	Local sports clubs (*n* = 3)

* *n* = total number of advocacy-related diary entries a Minister had during the period of time they held a health portfolio.

**Table 5:** Specific nutrition related issues documented in health ministers’ diaries (January 2013–June 2018).

Minister	Issue	Interest Group	Date
**QLD Health Minister ALP**	School Breakfast Program	YMCA	August 2015 September 2016
	Childhood Obesity	Happy Health Kids NFP	November 2015
	Cooking Skills	Jamie’s Ministry of Food Program	March 2016
	Diabetes prevention	Diabetes QLD	Oct 2017
**Health Minister QLD LNP**	Anti-obesity program	None	October 2013
	Junk food advertising	None	March 2013 April 2013
	Anti-obesity multicultural communities	Ethnic Communities Council of Queensland	March 2013
	Front-of-pack food labelling	None	December 2013
	Cooking Skills	Jamie’s Ministry of Food Program	May 2014
	Cooking skills	Queensland Country Women’s Assoc.	December 2014
**Health Minister NSW**	Cooking Skills	Jamie’s Ministry of Food Program	June 2015
	Aboriginal Nutrition	Newcastle University	August 2015 July 2016
	National Nutrition Week	Nutrition Australia	July 2017
	Healthy Choices in Health Facilities Policy	Australian Beverages Council	July 2017
**NSW Assistant Health Minister**	Obesity	Obesity Support Council	September 2014
	Food preservatives	Citizen	October 2015
